# Amplifying similarity to promote college STEM instructor–student mentoring relationship quality: a cluster randomized trial

**DOI:** 10.3389/feduc.2023.1293885

**Published:** 2023-11-22

**Authors:** Wenyi Du, Hyewon Lee, Nicole A. Broderick, Cristian Cervantes Aldana, Mica Estrada, Jo Handelsman, Natalia Maldonado, Sarah Miller, Megan S. Patterson, Perla Sandoval, Paul R. Hernandez

**Affiliations:** 1 Department of Teaching, Learning, and Culture, Texas A&M University, College Station, TX, United States; 2 Department of Biology, Johns Hopkins University, Baltimore, MD, United States; 3 Department of Social and Behavioral Sciences, University of California, San Francisco, San Francisco, CA, United States; 4 Department of Plant Pathology, University of Wisconsin-Madison, Madison, WI, United States; 5 Wisconsin Institute for Discovery, University of Wisconsin–Madison, Madison, WI, United States; 6 Department of Health Behavior, Texas A&M University, College Station, TX, United States

**Keywords:** college, STEM, mentor, instructor–student relationships, similarity, intervention

## Abstract

**Introduction:**

Despite numerous (co)curricular efforts, diversifying the Science, Technology, Engineering, and Mathematics (STEM) research workforce remains challenging and large segments of the U.S. population continue to be underrepresented. Promoting instructor–student mentoring relationship quality is a potentially important mechanism to support biomedical workforce diversity, as relationship quality has been positively associated with learning and persistence. We tested the impact of a “Creating Birds of a Feather” (CBoaF) intervention designed to promote perceptions of shared similarities (psychological similarity), which in turn should promote instructor-student mentoring relationship quality.

**Methods:**

This pretest-posttest cluster randomized controlled trial was conducted with a large and diverse sample of instructors (*J* = 15) and the undergraduates (*N* = 567) enrolled in biological course-based undergraduate research experience courses at 13 universities across the U.S.

**Results:**

Multilevel modeling results indicated that the intervention effect on undergraduates’ perceptions of psychological similarity was moderated by pretest psychological similarity. That is, among classes with low levels of similarity at pretest, the intervention group developed stronger perceptions of posttest psychological similarity than the control group, but there were no between group differences in classes with high levels of similarity at pretest. Furthermore, the intervention exhibited a positive indirect effect on posttest instructor–student mentoring relationship quality through posttest psychological similarity.

**Discussion:**

These findings highlight the potential of the CBoaF intervention to enhance undergraduate perceptions of instructor-student psychological similarity, subsequently leading to improved instructor-student mentoring relationship quality. These insights have significant implications for initiatives that aim to promote diversity and inclusion in the STEM research workforce by emphasizing the cultivation of psychological similarity between students and instructors.

## 1 Introduction

Promoting a diverse and inclusive environment in the Science, Technology, Engineering, and Mathematics (STEM) academics and workforce remains a key challenge and national priority. Recent studies have revealed a notable trend of high attrition among women and individuals from underrepresented racial/ethnic minority groups (URMs) in biomedical research careers (De Brey et al., 2019). Despite women’s remarkable achievement in surpassing men in college degree acquisition, comprising 57% of all bachelor’s degrees, they continue to be underrepresented in the majority of STEM fields at all levels of post-secondary education and in the STEM workforce (Snyder et al., 2019; National Center for Science and Engineering Statistics, 2023). Furthermore, even among those who initially intend to pursue STEM careers, women exhibit higher rates of departure from STEM fields compared to men during the secondary and post-secondary stages of education (Astorne-Figari and Speer, 2018). Similarly, URM students are highly underrepresented in biomedical fields; for example, although African American individuals constitute approximately 13% of the U.S. population, they account for just 7.8% of undergraduate degree recipients and 4.1% of doctoral degree recipients in the biomedical sciences field (National Center for Science and Engineering Statistics, 2023). It is increasingly clear that positive interactions with faculty members inside and outside the classroom are key to supporting student success in STEM – particularly for students from underrepresented groups (Hurtado et al., 2011; Ceglie, 2021). Consequently, it is imperative to gain a deeper understanding of the social and contextual factors that influence positive instructor-student interactions, as well as their impact on social integration into the biomedical community (Valantine and Collins, 2015; National Institutes of Health, 2019). The aim of this study was to experimentally test the impact of an intervention designed to improve instructor-student mentoring relationships in college biology course-based undergraduate research experiences (CUREs) focused on antibiotic discovery (Hurley et al., 2021).

Course-based undergraduate research experiences are an important classroom context in which to study mentoring, as CUREs are increasingly prevalent in undergraduate biological education (Auchincloss et al., 2014). In recent years, there has been a concerted effort to study and enhance mentoring experiences for undergraduate engaged in CUREs (Shanahan et al., 2015), which depart from traditional instructor-centered lectures or traditional laboratory experiments by emphasizing authentic student engagement scientific practices, scientific discovery in areas of interest to the wider scientific community, and collaboration through mentorship. Strategies for effective mentoring in CUREs include faculty availability, community building, addressing student needs, participation in the broader research community, and understanding students’ conditions in research programs (Pita et al., 2013). Extensive research has demonstrated the value of CUREs in fostering active learning, improving student well-being, solidifying a scientific identity, and positively influencing publication and career trajectories; however, the profound power of CUREs lies in the instructor/mentor-student dynamic (Linn et al., 2015; Ahmad and Al-Thani, 2022). Both access to a CURE mentor and effective mentoring from CURE instructors not only deepens undergraduates’ scientific understanding but also guides them in developing a distinct scientific identity and sustaining their persistence in research careers (Lopatto, 2004; Aikens et al., 2016; Joshi et al., 2019).

### 1.1 Theoretical framework

Over the last few decades, a variety of theoretical frameworks have been leveraged to better understand the STEM persistence gaps (Martín-Páez et al., 2019; Theobald, 2021). Among them, the Tripartite Integration Model of Social Influence (TIMSI) provides a strong theoretical framework to understand and address how and why people integrate into educational and professional communities from a social influence perspective (Estrada et al., 2011, 2018). TIMSI suggests that people socially integrate into academic communities through three distinct, but interrelated, social influence processes: self-efficacy (i.e., following regulations and norms to acquire rewards and prevent penalties in scientific community), identity (i.e., developing a social identity that encompasses the scientific community’s undertakings), and internalization of community values (i.e., embracing and disseminating the values upheld by the scientific community) (Kelman, 2006; Estrada et al., 2011). Furthermore, TIMSI indicates that members of the scientific community (e.g., faculty mentor) can act as key social influence agents to promote (or hinder) students’ integration into their scientific community through the provision of support that leads to gains in self-efficacy, identity, values, and ultimately student’s persistence in STEM fields (Kelman, 2006; Estrada et al., 2011, 2018; Hernandez et al., 2020). Mentoring relationships, which we define as a supportive relationship between an individual with more experience (mentor) and an individual with less experience (mentee) with the goal of advancing the mentee’s personal and professional growth (Crisp and Cruz, 2009; Hernandez et al., 2017), have been a central focus in prior TIMSI research on social integration into the scientific community. Prior tests of the TIMSI framework have shown robust evidence linking the quality of faculty-student mentorship support to gains in the TIMSI social influence process (i.e., efficacy, identity, and values) and persistence in undergraduate co-curricular research contexts (Hernandez et al., 2020). For example, a recent longitudinal study with African American and Hispanic undergraduates in STEM majors found that faculty mentor support promoted scientific self-efficacy, identity, and values in college, which in turn predicted persistence in STEM 4 years after graduation (Estrada et al., 2018). Although prior tests of the TIMSI framework provide compelling evidence of the socializing power of faculty-student mentorship, two critical gaps remain. First, the TIMSI framework has not yet been tested in undergraduate classroom contexts, focusing on the interactions between instructors and students. Second, less is known about the factors that promote instructor-student mentorship support and relationship quality within classroom contexts (such as CUREs). Therefore, in the sections below we briefly review (a) mentorship theory, (b) instructor-student relationship theory, and (c) empirical evidence on factors linked to the quality of mentoring relationships in college STEM contexts.

### 1.2 Mentoring in college STEM contexts

**FIGURE 1:**
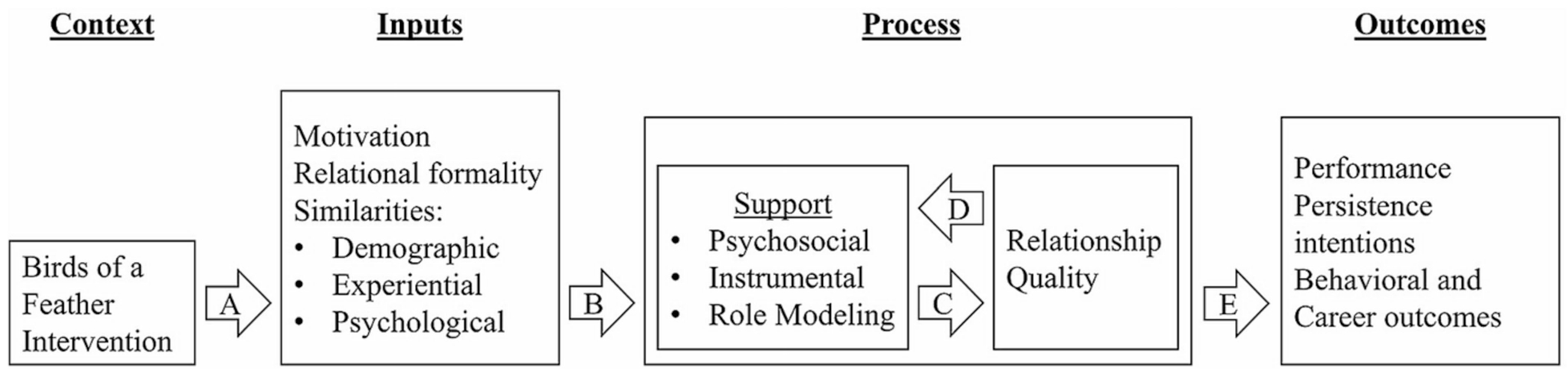
The conceptual model was adapted from Eby et al. (2013).

Understanding the factors that promote high-quality mentoring relationships, as well as the role that mentoring plays in promoting students’ engagement and success in college STEM fields can provide valuable insights for educational and STEM workforce stakeholders (National Academies of Sciences Engineering and Medicine, 2019). Eby et al. (2013) proposed the *Process Oriented Model of Mentorship* (POMM) that describes the inputs to, processes of, and outcomes from mentoring relationships based on a multidisciplinary meta-analysis of 173 empirical studies of mentoring in youth (K-12), college, and workplace contexts (Figure 1 shows our adapted and simplified version of the POMM) (Eby et al., 2013). Briefly, the POMM (consistent with TIMSI) suggests that mentoring relationship processes influence the mentee’s motivation, learning, and persistence (Figure 1, Path E; Eby et al., 2013). Mentoring processes are defined in terms of least three support functions: *Psychosocial support* through providing the mentee acceptance, counseling, and trust, *instrumental support* through practical assistance, apprenticeship, coaching, and sponsorship, and *role modeling support* by demonstrating the relevance and impact of their work, and helping mentees envision their own potential for similar achievements (Kram, 1985; Ragins and McFarlin, 1990; Tenenbaum et al., 2001). Meta-analytic findings indicate that mentoring support and relationship quality were exhibited small positive correlations with outcomes such as sense of belonging, learning, and persistence intentions (*ρ* range: |0.01| – |0.41|, median *ρ* = |0.22|; Eby et al., 2013). Consistent with the meta-analytic findings, recent research on mentoring in college STEM contexts found that high-quality mentoring support promoted small-to-moderate gains in STEM belonging, identity, interest, self-efficacy, persistence intentions, values, and well-being (Estrada et al., 2022; Saw et al., 2022; Du et al., 2023; Kuchynka et al., 2023). Furthermore, STEM undergraduates’ psychological similarity with their faculty mentor has a moderate-to-strong positive association with relationship quality and mentor-mentee relationship satisfaction (Hernandez et al., 2017, 2023; Pedersen et al., 2022).

#### 1.2.1 Factors that influence the mentoring relationship quality

Of particular interest for the current study, the POMM suggests that mentor and mentee characteristics (e.g., motivation, demographics [i.e., race, gender]), as well as mentor-mentee similarities should influence the quality of mentor support (Figure 1, Path B). Mentor-mentee similarities include: *demographic* or surface-level similarities such as race or gender, *experiential* similarity such as common educational, career, and life experiences, and *psychological * or deep-level similarity, involving shared attitudes, beliefs, and values (Harrison et al., 1998; Sánchez et al., 2005; Harden et al., 2009). Importantly, meta-analytic evidence indicated that psychological similarity was the only type of similarity that positively and moderately strongly correlated with mentorship support and relational satisfaction across studies (*ρ* range: 0.38–0.60, *ρ* rho = 0.49) (Eby et al., 2013; Ghosh, 2014).

Unsurprisingly, psychological similarity appears to be a primary driver of mentoring relationship quality. Laboratory based experimental social psychological research has found that social connections become stronger when individuals perceive shared characteristics (Montoya et al., 2008). Consistent with the similarity attraction paradigm, people tend to develop a preference for those who resemble themselves, and even minor similarities, such as attitudes, have the potential to enhance communication and interpersonal dynamics, which can in turn foster the development of attraction, liking, and positive relationships (Byrne, 1971). For example, laboratory experiments showed that individuals with matching initials exhibit improved collaborative performance (Polman et al., 2013), and individuals are more inclined to engage in helpful behaviors towards others who share the same birthday (Burger et al., 2004). In addition, research indicates that early perceptions of psychological similarity can moderate future mentor-mentee social interactions and relationship building behaviors (Hernandez et al., 2023). Thus, psychological similarity may be an important target for interventions designed to improve mentoring support and thereby promote social integration and persistence in STEM fields (Figure 1, Path A). Despite strong evidence linking psychological similarity with mentorship support, few studies in the mentoring literature have experimentally tested interventions to promote psychological similarity (Hernandez et al., 2023). We can, however, gain insight into potential factors and interventions to promote psychological similarity from the experimental instructor-student relationship (ISR) quality literature (Robinson et al., 2019).

Both experimental research and the mentoring literature provide compelling evidence demonstrating the manipulability of psychological similarity. Gehlbach et al. (2016) developed a procedure known as “Creating Birds of a Feather” (CBoaF), in which teachers and students complete interest surveys and are presented with their shared similarities as a means to enhance perceptions of similarity, improve ISR quality, and ultimately promote learning in high school settings (Gehlbach et al., 2016). The intervention demonstrated promising results by boosting teacher and student perceptions of similarity, enhancing the quality of their relationship and ultimately positively impacting student grades. Building upon this concept, a recent experimental study randomly assigned exposure to the CBoaF procedure with students and instructors in introductory college courses at a single university, with the goal of promoting ISR and student learning (Robinson et al., 2019). The results indicated that the CBoaF intervention resulted in small gains in psychological similarity but did not impact ISR or learning. However, the study also indicated that larger enrollment courses were associated with lower student perceptions of similarity with their instructor (Robinson et al., 2019). Similarly, a recent longitudinal quasi-experimental study found a college research mentoring program infused with activities designed to highlight shared similarities promoted psychological similarity between faculty mentors and students, which in turn promoted high-quality mentoring support (Hernandez et al., 2023). Given the inconsistent pattern of results across studies, more work is needed to understand for whom and under what circumstances CBoaF-type procedures induce both psychological similarity and relationship quality between instructors and students in college STEM classroom contexts.

### 1.3 Current study

**FIGURE 2:**
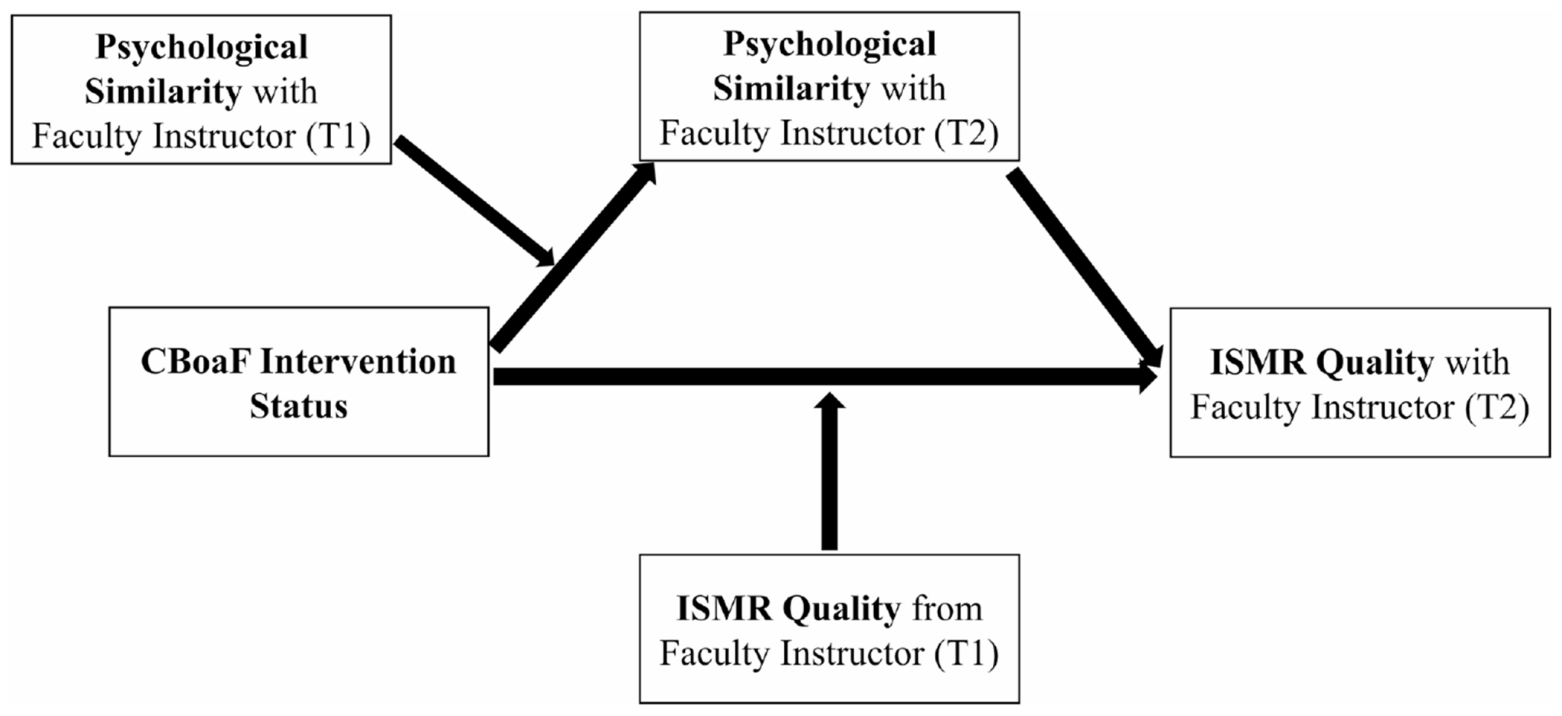
Conceptual statistical moderated mediation model linking the creating birds of a feather intervention with instructor-student mentoring relationship quality. CBoaF, creating birds of a feather; ISMR, instructor–student mentoring relationship.

Though evidence hints at the importance of ISR in STEM contexts, such relationships have been under-explored from a mentorship perspective (hereafter called as instructor-student mentoring relationship; ISMR). Accordingly, it is critical to examine ISMR to help students maximize their mentorship experiences thereby effectively pursuing their degrees in STEM. Guided by the TIMSI model and prior evidence in the mentoring and ISR literatures, we aimed to experimentally test the main and moderated impacts of a CBoaF intervention on (a) student perceptions of psychological similarity with their instructors and (b) the quality of the instructor-student mentoring relationship in a large and diverse sample of college instructors and students in biomedical courses (Figure 2). Furthermore, this study aimed to identify if the CBoaF intervention exhibited an indirect influence on IMSR through students’ perceived similarity (Figure 2). Specifically, this study implemented a cluster randomized pretest-posttest research design to address the following research questions:

1. Does exposure to the CBoaF intervention promote posttest student perceptions of similarity with and instructor-student mentoring relationship quality from the faculty instructor, while accounting for initial similarity or support?
2. Do initial similarity or support moderate the effect of the CBoaF intervention on posttest similarity or ISMR quality?
3. Is the effect of the CBoaF intervention on posttest ISMR quality mediated through posttest psychological similarity?

## 2 Methods

### 2.1 Participants

The current study used data from a larger longitudinal study of faculty and undergraduate mentoring relationships and career outcomes in biomedical science fields. The data were collected from 986 undergraduates clustered within 15 biological science college classrooms (seven control, eight experimental) across 13 universities in the U.S. Participants who did not respond to at least one of the outcomes were removed from the analytic sample resulting in a final sample of 505 students clustered within 15 instructors. Among students, nearly half self-identified as women and over one-third identified as being from one-or-more racial/ethnic minorities groups in STEM (Table 1). Regarding the faculty members, most self-identified as women and White (Table 1). Follow-up comparisons indicated there is no difference between students included in or excluded from the analytic sample in terms of intervention condition (*χ^2^*[1] = 1.34, *p* = 0.25).

### 2.2 Procedure

Faculty instructors in the biological sciences were recruited from a national network dedicated to training faculty to incorporate course-based research experiences. Instructors were randomly assigned to either the experimental condition (CBoaF intervention) or the control condition. In addition to receiving training in the course-based research experience, faculty in the experimental condition completed the Creating Birds of a Feather (CBoaF) survey and agreed to have their students in the research-based courses participate in the same activities. Faculty members assigned to the control condition received the standard course-based research experience training without any additional activities.

Students enrolled in the course-based research experience class were assigned to the same condition (experimental or control) as their faculty instructor (i.e., cluster random assignment). Students enrolled in the course-based research experience course were invited to participate in the study through emails distributed by their instructors. The students completed a pretest survey at the beginning of the semester (T1) and a posttest survey at the end of the semester (T2). They received a $10 incentive for completing each survey. The student participants were recruited across four cohorts: Fall 2020, Spring 2021, Fall 2021, and Spring 2022. The study received approval from the local Institutional Review Board (IRB#19-28867).

#### 2.2.1 Creating birds of a feather intervention

At the end of the pretest survey (T1), students and instructors in the CBoaF intervention group were given a “getting to know you” questionnaire (Robinson et al., 2019), using Qualtrics survey platform. This section asked multiple-choice questions about their interests (e.g., “On a day off school and/or work, which of the activities are you most likely to do?”), personal lives (e.g., “Do you have a family member or close friend who is in the military?”), and values (e.g., “The most important quality in a friend is…”). Upon completion, students received a personalized email to discover three areas of similarity (one per category) with their instructor. Finally, to help internalize the similarities, students completed a few brief questions about their commonalities and how these similarities might be leveraged later in the semester.

### 2.3 Measures

The collection of data in our study included demographic information from both students and faculty, which was obtained through a pre-test survey (T1). Additionally, measures of psychological similarity and mentoring quality were assessed on both pre- and posttest surveys (T1 and T2). Composite scores were generated for each measure by averaging the items, with higher mean scores indicating a greater level of the measured construct.

#### 2.3.1 Psychological similarity

To evaluate the psychosocial similarity between instructors and students, we employed a five-item scale adapted from prior research (Ensher and Murphy, 1997). Participants were asked to rate their level of agreement with statements pertaining to their alignment with their instructor (e.g., “My instructor and I see things in much the same way.”) using a scale ranging from 1 (strongly disagree) to 5 (strongly agree). Internal consistency reliability was acceptably high at each timepoint (Table 2).

#### 2.3.2 Instructor–student mentoring relationship quality

We assessed ISMR quality with five items taken from previously validated scales to capture global mentoring quality, which included psychosocial support, career support, role modeling support and relationship satisfaction (Dreher and Ash, 1990; Hoyt et al., 2012). The items were modified to focus on the mentorship relationship with their course instructor. Participants were asked to rate their agreement with statements reflecting the mentoring quality they received from their instructor (e.g., “My instructor has conveyed empathy when I have discussed my concerns or feelings with them.”) using a scale ranging from 1 (not at all) to 5 (to a very large extent). Internal consistency reliability was acceptably high at each timepoint (Table 2).

#### 2.3.3 Student and faculty instructor demographics and gender-match

Participants self-reported demographic information, including gender identity and race/ethnicity. Gender identity for participants was dummy coded, with women serving as the reference group. Additionally, race was dummy coded to reflect participants’ minority status in the STEM field (1 = racial minority, 0 = racial majority, e.g., White & Asian). Furthermore, we used information on gender identity to derive a dummy coded variable to reflect the instructor-student gender-match status (1 = same gender, 0 = different genders).

#### 2.3.4 Experiment condition status

A binary variable was generated to indicate the participants’ group assignment. A value of zero represents the control group, while a value of one indicates membership in the experiment (CBoaF) group.

### 2.4 Plan of analysis

We conducted a series of moderated regression models to address our research questions, accounting for the nested nature of the data and research design (i.e., cluster randomization and students nested within instructors) using cluster robust standard error estimation. Specifically, posttest psychological similarity was regressed on CBoaF group status (RQ1), pre-test psychological similarity (centered), a pre-test psychological similarity-by-experimental group status interaction term (RQ2), and a set of control variables (listed below). Similarly, posttest ISMR quality was regressed on CBoaF group status (RQ1), pre-test ISMR (centered), a pre-test ISMR-by-experimental group status interaction term (RQ2), posttest psychological similarity (centered) (RQ3), and a set of control variables. The control variables included student and instructor gender identity, a student by instructor gender identity interaction term (to capture gender-match effects), and course enrollment size. Finally, a mediation model was estimated to assess the indirect effect of the CBoaF intervention on ISMR quality through psychological similarity using a bootstrapping procedure with 3,000 iterations to estimate confidence intervals around the indirect effect (RQ3). The regression models were conducted using OLS regression with cluster robust standard error estimation via the SUREG command in Stata v.17 (Stata, 2021). Power analyses were performed to determine the minimum detectable effect size with a conventional power threshold (power = 0.80), given the sample size in our study in G*Power v3.1 and Monte Carlo Power Analysis for Indirect Effects (Faul et al., 2009; Schoemann et al., 2017). The power analyses revealed that our study was adequately powered to detect a small main effect of the CBoaF intervention (*R^2^* = 0.015) and a CBoaF intervention by pretest moderation effect (*R^2^* = 0.029), as well as a small standardized indirect effect (*β_a × b_* = 0.002).

## 3 Results

Prior to conducting formal tests, we examined the descriptive and correlational statistics (Tables 2, 3). The analysis revealed that compared to CBoaF classes, control group classes reported somewhat higher average levels of psychological similarity and instructor-student mentoring relationship quality at pretest. These pre-test differences indicated that cluster random assignment did not result in equivalent groups at baseline. Therefore, pretest measures of psychological similarity and ISMR quality were included in all models to account for pretest classroom level differences. In addition, consistent with our expectations psychological similarity was positively correlated with ISMR quality at both pre- and posttest.

**FIGURE 3:**
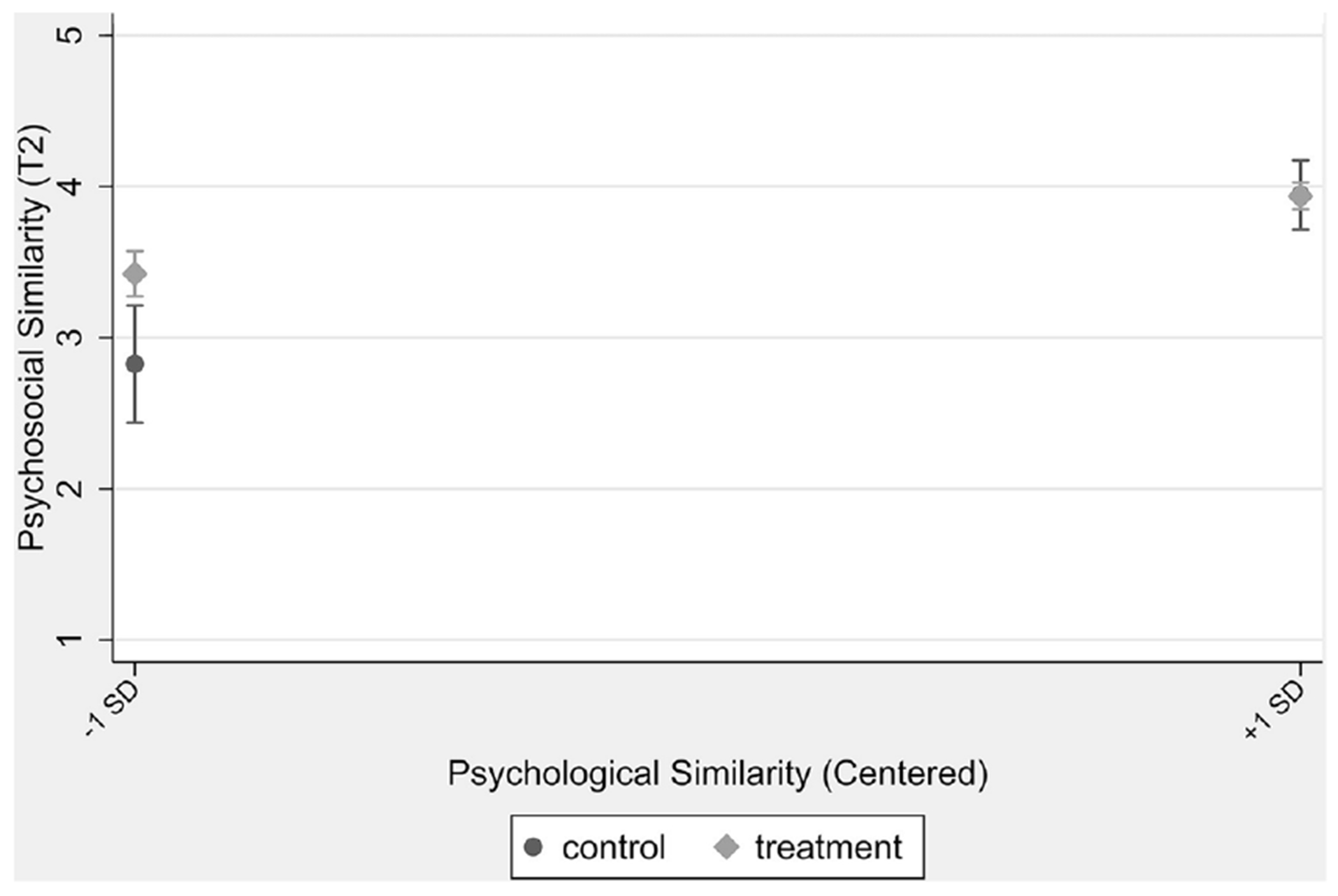
Effect of CBoaF intervention on psychological similarity at posttest (T2), moderated by psychological similarity at pretest (T1, Centered).

Next, we used multiple regression with cluster robust standard errors to assess the impact of the CBoaF intervention on student perceptions of psychological similarity with and ISMR quality from their biology course instructor at posttest (RQ1), as well as if pretest psychological similarity or ISMR quality moderated the intervention effect (RQ2; detailed in Plan of analysis). Concerning psychological similarity, the results revealed no main effect of the CBoaF intervention, however, the intervention effect was moderated by pretest psychological similarity (Table 4). The results indicated that among students who expressed low perceptions of psychological similarity at pretest (Figure 3, groups at −1SD), the CBoaF intervention group reported higher posttest psychological similarity than the control group. By contrast, among students who expressed high psychological similarity at pretest (Figure 3, groups at +1SD), the CBoaF and control groups perceived equally high levels of similarity with their instructor. Concerning ISMR quality, the results revealed that neither a main nor a moderated effect of the CBoaF intervention (RQ1 and RQ2).

**FIGURE 4:**
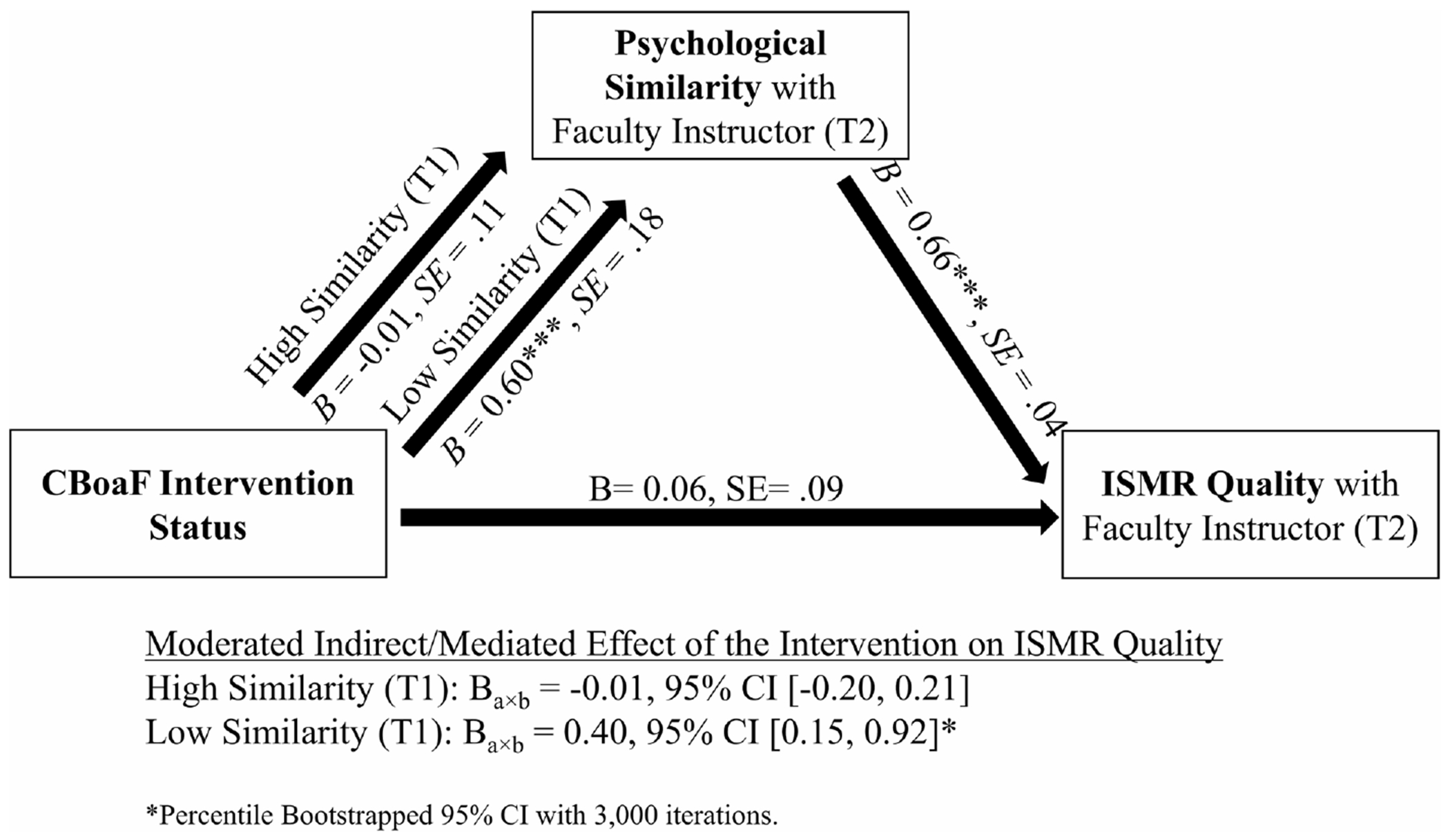
Summary of the moderated mediation results linking the CBoaF intervention with ISMR quality through psychological similarity. CBoaF, creating birds of a feather; ISMR, instructor–student mentoring relationship.

Finally, we tested the indirect effect of the CBoaF intervention on students’ perception of ISMR quality through psychological similarity at posttest (RQ3). Given the CBoaF by pretest psychological similarity moderation effect, we performed a moderated mediation analysis using the bootstrapping procedure described above. As shown in Figure 4, the results revealed that the CBoaF intervention had a positive impact on ISMR quality through psychological similarity; however, this positive mediated effect only held for students who expressed low perceptions of psychological similarity at pretest. That is, among students who initially perceived little similarity with their instructor, the CBoaF intervention boosted their perceptions of similarity and this in turn promoted receiving higher levels of instructor-student mentoring relationship quality.

## 4 Discussion

National statistics in the U.S. show that many STEM fields have yet to achieve the goals of creating a robust, diverse, and inclusive workforce (National Center for Science and Engineering Statistics, 2023). However, research indicates that positive social interactions between undergraduates and college STEM faculty, instructors, and/or mentors has the potential to promote students’ social integration into and persistence in STEM fields – particularly for students from historically underrepresented groups (Luo et al., 2022; Pedersen et al., 2022; Du et al., 2023). Moreover, research in the mentoring and instructor-student relationship literatures indicate that student perceptions of psychological similarity may be a useful target for intervention, as perceived similarity has beneficial effects on forming high-quality relationships and social integration (Estrada et al., 2022; Marmet, 2023). This study extends prior research by testing the main (RQ1), moderated (RQ2), and mediated (RQ3) impacts of a “Creating Birds of a Feather” intervention aimed at promoting students’ perceptions of psychological similarity and mentoring relationship quality with their biology course instructor using a pre-post cluster randomized design.

Our data revealed an interesting and nuanced pattern of findings related to impacts on student perceptions of psychological similarity with their course instructor. First (and inconsistent with prior research among college students), the CBoaF intervention did not universally boost student perceptions of psychological similarity with their instructor at posttest (RQ1). We suspect this was due to imbalance after cluster randomization. That is, although our cluster randomized design resulted in an equal number of courses assigned to intervention and control conditions, the CBoaF classes happened to have much larger enrollment size on average than the control classes. Prior research indicates that students in larger enrollment classes can have lower perceptions of similarity with their instructors than those in small enrollment courses (Robinson et al., 2019). And consistent with prior research, we found that larger course enrollment size was associated with lower perceptions of similarity. Second (and partially consistent with our expectations), the CBoaF intervention impact was moderated by first impressions of similarity – that is the intervention boosted perceptions of psychological similarity *among students who initially expressed low levels of perceived similarity* (RQ2). This finding was consistent with prior work indicating that early impressions of similarity can influence the link between mentor-mentee social interactions and the mentee’s perceptions of the relationship (Zheng et al., 2021).

In addition, our data revealed a clear pattern of finding related to CBoaF impacts on instructor-student mentoring relationship quality. We found that the CBoaF intervention did not directly impact student perceptions of ISMR quality, which was consistent with prior experimental research with college students (Robinson et al., 2019). This finding is consistent with mentoring models, such as the POMM, which indicate that only certain inputs or antecedents directly influence the quality of mentoring relationships (e.g., motivation, similarity). In addition, we found that student perceptions of psychological similarity with their instructor promoted their perceptions of instructor-student mentoring relationship quality, which is consistent with robust research in the mentoring literature (Eby et al., 2013). As a whole, it was clear that the CBoaF intervention had a positive *indirect* effect on student perceptions of ISMR quality through its influence on their perceptions of similarity with their instructor (RQ3). That is, among students with initially low impression of the similarity, the intervention boosted final impressions of similarity, which in turn promoted their perceptions of ISMR quality.

### 4.1 Limitations, future research, and implications

While this study significantly contributes to our knowledge of instructor–student mentoring in undergraduate biomedical science contexts, we acknowledge several limitations. First, our cluster randomized procedures did not result in completely unbalanced experimental and control conditions, as evidenced by differing class enrollment sizes. Unequal sample sizes can lead to larger groups exerting a stronger influence on the overall analysis compared to smaller groups (Raudenbush, 1997; Konstantopoulos, 2010). Consequently, there was a potential for our findings to be more influenced by the larger CBoaF group. However, we aimed to mitigate this limitation by introducing statistical controls, such as the students’ initial status at pretest and the course enrollment variable, thereby accounting for unbalanced sample sizes and other pre-existing differences between the groups that could have influenced the outcomes. We believe that our approach minimizes the potential bias introduced by the uneven sample sizes. To address this limitation and strengthen the validity and generalizability of the findings, future research should strive to examine the effects across a larger and more balanced sample of classes. A further limitation of the current study concerns our global assessment of instructor-student mentoring relationship quality (e.g., psychosocial support, instrumental support) did not allow us to disentangle the effect of the intervention on specific facets of the relationship quality. Future research examining more granular types of support can improve our understanding of the impacts of CBoaF intervention on types of support. Additionally, extending the study to encompass a broader range of university settings would help determine if the observed intervention effects hold true in different STEM field contexts. Further, research is needed to examine the long-term effects beyond the duration of a single course and to test the psychosocial mechanisms proposed by the TIMSI model, which may mediate the relationship and provide a deeper understanding of whether and why the intervention may have long-term impact.

There are several implications of this research. First, taken together, these findings indicate that student perceptions of similarity appear to be quite sensitive to the college classroom environment. That is, larger enrollment classes appear to dampen first impressions of similarity with instructors, which in turn could have negative effects on the level of support they perceive from their instructor, and ultimately undermine their social integration. Therefore, instructors and designers of larger enrollment courses should consider carefully how to create learning environments that promote mutually satisfying connections with the instructor and peers.

Second, larger enrollment classes present an opportunity for CBoaF-type interventions targeting perceptions of similarity. The CBoaF intervention was inexpensive, scalable, and relatively easy to automate and integrate into the classroom setting. Our research indicates that even in this potentially fraught college environment, the CBoaF intervention boosted the perceptions of similarity among those with the weakest first impression of similarity. It is notable that students with weak first impressions of similarity with their instructor are not easily identified through proxies such as gender, race, or generational status (at least in our data). Perceptions of similarity, more so than any other factor we measured, determined the quality of the mentoring relationship students experience.

More broadly, these findings indicate that psychological similarity, which is typically thought of as a key input or antecedent to mentoring relationship quality (Eby et al., 2013), is itself a manipulable target for intervention. And interventions that can successfully promote psychological similarity can have beneficial downstream effects on relationship quality and related outcomes (Eby et al., 2013; Hernandez et al., 2017; Pedersen et al., 2022).

## 5 Conclusion

Our study contributes to the understanding of the potential impact of an intervention aimed at promoting student-instructor relationship (ISMR) quality within the college biological science classroom contexts. Specifically, we implemented a pretest-posttest cluster randomized research design to test the impact of a “Creating Birds of a Feather” intervention on undergraduate students’ perceptions of similarity with their instructor as well as their perceptions of their instructor-student mentoring relationship. The results demonstrate the nuanced nature of the intervention’s effects on perceived similarity and ISMR quality, while shedding light on the role of students’ initial similarity. These findings have implications for designing effective mentoring interventions tailored to individual needs and enhancing instructor-student relationships in the field of biomedical education.

## Figures and Tables

**TABLE 1:** Faculty and student demographic characteristics as a function of CBoaF intervention status.

	Condition	
	Control	CBoaF
Student (*N* = 505)		
Gender identity		
Women^a^	26 (41%)	210 (48%)
Men	16 (25%)	97 (22%)
Transgender/Non-binary	0 (0%)	5 (1%)
Prefer to not say	22 (34%)	129 (29%)
Race/Ethnicity		
Prefer to not say	0 (0%)	3 (<1%)
White^b^	42 (66%)	144 (33%)
Hispanic	6 (9%)	124 (28%)
Black	5 (8%)	20 (4%)
Asian^b^	4 (6%)	82 (19%)
American Indian	0 (0)	2 (<1%)
Middle Eastern	1 (1%)	10 (2%)
Native Hawaiian	0 (0%)	4 (1%)
Other	1 (1%)	4 (1%)
Multi-racial/ethnic	5 (8%)	48 (11%)
Total	64	441
Faculty (*J* = 15)		
Gender identity		
Women	5 (71%)	5 (63%)
Men	2 (29%)	3 (37%)
Race/Ethnicity		
White	7 (100%)	7 (88%)
Hispanic	0 (0%)	1 (12%)
Total	7	8

**TABLE 2:** Summary of descriptive statistics and correlation among variables in the models (*N* = 505).

Variables	1	2	3	4	5	6	7	8
1. Mentoring quality (T2)	*0.88*							
2. Mentoring quality (T1)	0.48*	*0.90*						
3. Psychological similarity (T2)	0.70*	0.35*	*0.86*					
4. Psychological similarity (T1)	0.39*	0.66*	0.40*	*0.88*				
5. CBoaF Status (1 = Exp.)	−0.17*	−0.15*	−0.15*	−0.16*	--			
6. Student gender (Man Status)	0.08	−0.01	0.03	0.03	−0.03	--		
7. Student gender (TB/P Status)	0.09*	0.09*	0.09*	0.08	−0.03	−0.36*	--	
8. Faculty gender (Man Status)	−0.10*	−0.19*	−0.01	−0.08	−0.06	0.12*	−0.34*	--
9. Course Enrollment Size	−0.27*	−0.20*	−0.26*	−0.19*	0.66*	−0.01	−0.19*	−0.23*

Italicized values on diagonal of the correlation matrix are coefficient alpha values. Student’s and faculty’s gender were dummy coded with women as the reference group. For student gender groups, Transgender, non-Binary, and Prefer not to say groups were combined (TB/P). T1, construct measured at time 1; T2, construct measured at time 2;

* *p* < 0.05,

** *p* < 0.01.

**TABLE 3:** Summary of descriptive statistics for the outcomes as pre- and posttest as a function of CBoaF intervention status.

	Control *N* = 64 (*n_j_* = 10.67)				CBoaF *N* = 441 (*n_j_* = 63.14)			
	*M*	SD	*S*	*K*	*M*	SD	*S*	*K*
Mentoring quality (T2)	4.09	0.72	−0.89	3.74	3.63	0.92	−0.45	2.56
Mentoring quality (T1)	3.92	0.71	−0.18	2.65	3.56	0.80	−0.30	2.93
Psychological similarity (T2)	3.96	0.73	−0.94	5.65	3.62	0.80	−0.33	3.59
Psychological similarity (T1)	3.83	0.64	0.15	2.37	3.49	0.73	−0.02	3.63

n_j_, average course enrollment size; M, mean; SD, standard deviation; S, skewness; K, kurtosis.

**TABLE 4:** Results of multilevel models predicting posttest psychological similarity and relationship quality.

Predictors	Estimates (*b*)	SE_Robust_	*p*
Psychological similarity (T2)			
Intercept	3.84	0.14	<0.001
Psychological similarity (T1)	0.77	0.10	0.62
CBoaF	0.30	0.13	0.02
CBoaF × Psychological similarity (T1)	−0.42	0.12	<0.001
Student man status (SMS)	−0.05	0.10	0.62
Student other gender status	−0.02	0.05	0.69
Faculty man status (FMS)	−0.12	0.07	0.08
SMS × FMS	0.17	0.15	0.23
Course enrollment size	−0.002	0.0003	<0.001
ISMR quality (T2)			
Intercept	3.85	0.07	<0.001
ISMR quality (T1)	0.30	0.05	<0.001
Psychological similarity (T2)	0.66	0.04	<0.001
CboaF	−0.06	0.09	0.51
CBoaF × ISMR quality (T1)	−0.01	0.09	0.91
Student man status (SMS)	0.20	0.05	<0.001
Student other gender status	−0.003	0.04	0.95
Faculty man status (FMS)	−0.13	0.04	0.001
SMS × FMS	−0.06	0.06	0.28
Course enrollment size	−0.001	0.0003	0.01

ISMR, instructor–student mentoring relationship. *N*_Students_ = 505, *J*_Faculty_ = 13. SE_Robust_, cluster robust standard errors. All continuous predictors were centered for these analyses.

## Data Availability

The data that support the findings of this study are openly available in the Texas Data Repository at https://doi.org/10.18738/T8/YFOZQ2.
